# Supplementary material for: Sequence- and Structure-Specific tRNA Dihydrouridylation by hDUS2
Source: ACS Cent Sci. 2024 Mar 12;10(4):803–12. doi: 10.1021/acscentsci.3c01382 (PMC11046453; doi:10.1021/acscentsci.3c01382)
Supplement: Supplementary file 2 — oc3c01382_si_002.pdf [file oc3c01382_si_002.pdf]

oc-2023-01382r.R1

Name: Peer Review Information for "Sequence- and structure-specific tRNA dihydrouridylation by hDUS2"

First Round of Reviewer Comments

Reviewer: 1

Comments to the Author

This is a cool chemical biology study taking the concept of covalent enzyme inhibition into the field of RNA modification. The probe design, building on 5-fluorouracyl and the idea to, after reduction to a dihydroacyl moiety, creating a more reactive halomethylketone for cysteine nucleophilic attack is creative and the strategy bears out. The authors convincingly show selectivity for their target enzyme (dihydrouridine synthase 2) with only a specific uridine out of (potentially) multiple ones being modified, and in a sequence (one nearby G) specific manner. The technology appears amenable for adaptation towards the other DUS enzymes and can be translated into activity-based probes, should the authors wish to go in that direction. The technology also allows, as is demonstrated in proof-of-concept studies, for the identification of DUS inhibitors with potential clinical relevance. The work is written and presented in a clear and crisp way, with a strong experimental and analytical foundation. I recommend acceptance as is.

Reviewer: 2

Comments to the Author

In this manuscript the authors study human dihydrouridine synthase 2 (hDUS2) and investigate its enzymatic activity and substrate specificity using novel activity-based probes. Previous work from the Kleiner Lab revealed that another human dihydrouridine synthase 2, namely DUS3L, modifies numerous RNA species on a transcriptome-wide scale. Although, the metabolic RNA labelling approach revealed possible modification sites, the detailed mechanisms governing the substrate specificity of human DUS enzymes remains largely unknown.

Here, the authors developed an activity-based chemical crosslinking method that can be applied to study RNA modifying enzymes like human DUS2. To investigate the hDUS2 substrate selectivity towards human tRNA molecules the authors first developed crosslinking warheads that specifically

and covalently links catalytic cysteine residue of hDUS2 to modifiable uridine of IVT tRNAs. This approach allowed to identify covalent adducts by LC/MS and to map the crosslinked sites on the tRNA and DUS2. The authors also carried out a systematic analyses of 11 IVT tRNA substrates and confirmed that the recognition of tRNA by hDUS2 enzyme is independent of the ASL – still requiring the acceptor stem and T-arm domains. The authors also determined that the selectivity of hDUS2 depend on residues neighboring the modifiable uridine, revealing a conserved guanidine (G19) residue next to the U20, which seems essential for the recognition of modification sites. Finally, the authors screen 5 commercially available EGFR kinase inhibitors, known to be modulating the hDUS activity.

I appreciate how the authors provide a comprehensive description of chemical crosslinking reaction including the experiments with hDUS2-C116A mutant, RNase treatment and all tested tRNA variants (without CCA, ACL or T-arm). The manuscript does not close the gap of missing structural information, which would allow to directly characterize the mechanism of tRNA recognition by human DUS enzymes. However, there is a significant amount of new information presented in this manuscript – on an important topic that is understudied. In summary, the manuscript is timely and original - I recommend publication in ACS Central Science after the following points have been addressed.

Major issues

1. I was surprised to see that the DUS2 C116A mutants still seems to show some modification activity (Suppl. Fig. 3). I am aware that the detected activity is still lower than for the wild type protein, but it still seems very surprising that this mutant is able to convert U to D at all – could the authors discuss this finding and provide possible alternative residues that can take promote the modification reaction in the absence of the catalytic cysteine residue? Did the authors test other active site mutants?

2. Suppl. Fig 4b, the gel of IVT BrUrd-Val tRNA clearly shows 2 lower bands, indicating other species of produced tRNA (3'truncations or not-properly folded tRNAs). Did the authors analyze all 11 produced tRNA and correlate the level of heterogenicity of IVT tRNA with the effect on crosslinking efficiency. It could mean that less stable tRNAs that have lower crosslinking efficiency (i.e. Tyr or Asn) are simply more heterogeneous. The quality of IVT tRNA preparations could influence conclusions made by authors. Did they consider synthesis of tRNAs or tRNAs fragments (i.e. without ACL) that would have only modified Br-Urd at position 20 instead of exchanging all Us? Modifying all U bases may have detrimental effect on tRNA folding and it would be necessary to more quantitatively assess the quality of the IVT tRNAs (e.g. Tm, DSL).

3. Fig.2b and Suppl. Fig.5,– crosslinking reactions were performed with the substantial molar excess of tRNA (1uM or 10uM) to protein (200nM) content (5-fold or 50-fold excess of BrU-tRNA-Val). This result suggests that the crosslinking reaction has low efficiency and indicates that BrU-tRNA is indeed not properly folded (see point above) and could be improved by specific modification of only U20 base, targeted by hDUS2. A similar observation was made in BrU-tRNA-Val titration (Suppl. Fig.7), as the authors state on p.7: "Crosslinking yields did not exceed ~50% despite using up to 75-fold excess of BrU-tRNA-Val-CAC, which is likely due to instability of the enzyme

during the reaction but could also reflect inefficiency in the crosslinking chemistry." Moreover, the upscaled experiment (Suppl. Fig. 8) for proteomics analysis have reached 95% crosslinking efficiency with the use of 2 µg of recombinant hDUS2 with 7.5 µg of BrUrd-modified tRNA-Val-CAC (3,75-fold excess of tRNA). Is it the same IVT BrU-tRNA-Val preparation? If not, it could mean that tRNA purity and folding varies between the preparation and should be clarified.

4. As the highest efficiency of crosslinking in Fig.3a-d was observed for tRNAGlu and tRNAVal, the optimal sequence recognition motif could be G18G19U20U20a – present in both tRNAs and not in the others tested. Similarly, the lack of dsRBD domain of hDUS2 doesn't abolish the crosslinking to both tRNAGlu and tRNAVal, indicating stronger interactions of the catalytic core with the G18G19U20U20a / G18G19U20 motif as the one present in tRNAGly and tRNALeu, which is efficiently modified by hDUS2 lacking the dsRBD (Fig. 3d, Suppl. Fig. 15). Moreover, the experiments with the modified oligonucleotides (shown on Suppl. Fig. 13), and with truncated tRNAs (Suppl.Fig. 14) indicate that substrate selection by hDUS2 requires tertiary tRNA structure determinants as deletion of T-arm or acceptor stem abolished crosslinking to hDUS2. Therefore, the "GU" motif seems not to be sufficient for governing the hDUS2 substrate selectivity. The authors could consider comparing the predicted 3D structures of all tested IVT tRNAs to conclude about DUS2-selective determinants in tRNAs. Foremost, I think the authors overstate the phrase "motif" and I would kindly ask the authors to down-tone this statements – on the one hand they show that whole domains of the tRNA are important and on the other hand they claim that the "motif" only consists of GU – this is inconsistent and confusing to the novice reader.

5. Related to the slight overstatement about the identified "motif", I would suggest to adjust the title – at the moment it is rather confusing and does not describe the major findings in the most clear way.

Minor points:

- Removal of the ASL doesn't seem to influence the interaction between DUS2 and tRNA as shown by activity-based crosslinking experiments (Fig3e,f) and all tested tRNAs harbor the GU motif. I would ask the authors to comment on the observed large variety in crosslinking efficiency between different tRNA species (Fig.3a,b and Fig3.c,d)? - also see points above.

- The authors claim that they have identified the minimal GU motif that governs the activity of hDUS2 enzyme. There are not enough direct experiments supporting this conclusion. The G19 is very conserved base in almost all human tRNA molecules. The lack of G preceding the modifiable U20 may lead to disrupted substrate recognition but does not influence the enzyme activity. Thus, the abstract sentence on p.2 should be rephrased: "We find that hDUS2 modifies U20 in the D loop of diverse tRNA substrates and identify a minimal GU motif within the tRNA tertiary fold required for directing its activity." Furthermore, the paragraph on page 15 seems way too speculative – "G19 is known to Watson-Crick base pair with C56 in the T loop23, forming a conserved tertiary interaction at the tip of the tRNA "elbow". It is likely that direct recognition of the G19:C56 pair in the context of this structure is responsible for setting the register of modification. Interestingly, mutational insertion of G at position 20 in either tRNA-Val-CAC or tRNA-Arg-ACG enables modification by

hDUS2 at position 20a. One possible explanation for this behavior is that G20 pairs with C56 in place of G19 leaving the overall elbow structure unperturbed." – please rephrase.

- I would suggest to label the RNA bases as U20 etc. instead of U20.

- The sentence in the introduction on p.4 asks for an explanation WHY the authors did not follow up on the previous results for DUS3L - "Despite the proposed mechanistic similarity among DUS enzymes, we found that 5-FUrd did not react appreciably with human DUS enzymes other than DUS3L. In addition, we did not characterize the nature of the 5-FUrd-DUS3L crosslink nor did we reconstitute DUS3L-RNA crosslinking in vitro.". Did the authors try to reconstitute the DUS3L-RNA in vitro and failed?

- Suppl. Fig. 11 and 12 – it would be very interesting to identify the crosslinking U base in tRNAMet as it may not be the U16. It could also be considered as an artefact since the crosslinking efficiency is very low – please comment.

- Suppl. Fig. 45 – please explain the double peak at the observed LC-MS analysis.

- Page 11 line 39 - typo "a similar same set of tRNAs"

Reviewer: 3

Comments to the Author

In this manuscript, Ji et al provide a series of experiments to test the substrate specificity of hDUS2. These approaches include mechanism-based crosslinking and oligonucleotide LC-MS/MS. Through their approach they were able to show the molecular determinants for substrate recognition and modification by hDUS2. Besides this, the authors further developed a screening approach to identify small molecule inhibitors of hDUS2, that is involved in human cancer. The manuscript is extremely well organized, very comprehensive and answers all the main questions raised in the initial hypothesis. Through the reading of the manuscript the reader is led to think about certain questions, as for example, how is substrate specificity of DUS2 in other tRNAs, or how can the authors ensure that BrUrd modification is not affecting the enzyme recognition. For all these questions, the authors provide answers with compelling evidence that are the result of the development and implementation of several very elegant strategies. The establishment of an oligonucleotide LC-MS platform to characterize the site of hDUS2 mediated formation on unmodified IVT tRNA substrate is another example of a very elegant methodology that can be applied to study many other tRNA modifications. In sum, this is a very good manuscript that, in my opinion, is mostly ready to be published. I only have 2 very minor remarks:

- Fig 2b is not mentioned in the text

- Maybe the text following the question "How does hDUS2 select a single U residue among the multiple possible U substrates within the D loop?" could be improved, as the introduction of a

question in the middle of the results section may be misleading. But this is more a matter of "taste" rather than a critique, and I leave it to the authors consideration.

Reviewer: 4

Comments to the Author

Kleiner and coworkers describe the use of a novel chemical tool set to understand the selectivity of human uridine to dihydrouridine tRNA modifications catalyzed by the hDUS2 tRNA modification enzymes. Previous work has established the biological importance of hDUS2 modifications and also possible disease relevance. Less understood has been the selectivity of these enzymes and exact biological role of such modifications, and its clear that, even more broadly speaking, novel tools for understanding and characterizing tRNA modifications are needed. Kleiner uses a clever mechanism based cross linking assay to better understand hDUS2 activity. They identify the necessary selectivity requirements for modification and even demonstrate that the tool could prove useful for screening drug interactions with the enzyme. Overall, it is technically very well executed. One question that came up during reading was the surprising result that Afatinib increases activity of hDUS2. This is mentioned but not commented on and I think its worth a brief discussion of what might be going on.

Author's Response to Peer Review Comments:

Reviewer(s)' Comments to Author:

Reviewer: 1

Recommendation: Publish in ACS Central Science without change.

Comments:
This is a cool chemical biology study taking the concept of covalent enzyme inhibition into the field of RNA modification. The probe design, building on 5-fluorouracyl and the idea to, after reduction to a dihydroacyl moiety, creating a more reactive halomethylketone for cysteine nucleophilic attack is creative and the strategy bears out. The authors convincingly show selectivity for their target enzyme (dihydrouridine synthase 2) with only a specific uridine out of (potentially) multiple ones being modified, and in a sequence (one nearby G) specific manner. The technology appears amenable for adaptation towards the other DUS enzymes and can be translated into activity-based probes, should the authors wish to go in that direction. The technology also allows, as is demonstrated in proof-of-concept studies, for the identification of DUS inhibitors with potential clinical relevance. The work is written and presented in a clear and crisp way, with a strong experimental and analytical foundation. I recommend acceptance as is.

Response: We thank the reviewer for their thoughtful and positive review of our manuscript.

Additional Questions:
Quality of experimental data, technical rigor: Top 5%

Significance to chemistry researchers in this and related fields: Top 1%

Broad interest to other researchers: Top 5%

Novelty: Top 1%

Is this research study suitable for media coverage or a First Reactions (a News & Views piece in the journal)?: No

Reviewer: 2

Recommendation: Reconsider after major revisions noted.

Comments:
In this manuscript the authors study human dihydrouridine synthase 2 (hDUS2) and investigate its enzymatic activity and substrate specificity using novel activity-based probes. Previous work from the Kleiner Lab revealed that another human dihydrouridine synthase 2, namely DUS3L, modifies numerous RNA species on a transcriptome-wide scale. Although, the metabolic RNA labelling approach revealed possible modification sites, the detailed mechanisms governing the substrate specificity of human DUS enzymes remains largely unknown.

Here, the authors developed an activity-based chemical crosslinking method that can be applied to study RNA modifying enzymes like human DUS2. To investigate the hDUS2 substrate selectivity towards human tRNA molecules the authors first developed crosslinking warheads that specifically and covalently links catalytic cysteine residue of hDUS2 to modifiable uridine of IVT tRNAs. This approach allowed to identify covalent adducts by LC/MS and to map the crosslinked sites on the tRNA and DUS2. The authors also carried out a systematic analyses of 11 IVT tRNA substrates and confirmed that the recognition of tRNA by hDUS2 enzyme is independent of the ASL – still requiring the acceptor stem and T-arm domains. The authors also determined that the selectivity of hDUS2 depend on residues neighboring the modifiable uridine, revealing a conserved guanidine (G19) residue next to the U20, which seems essential for the recognition of modification sites. Finally, the authors screen 5 commercially available EGFR kinase inhibitors, known to be modulating the hDUS activity. I appreciate how the authors provide a comprehensive description of chemical crosslinking reaction including the experiments with hDUS2-C116A mutant, RNase treatment and all tested tRNA variants (without CCA, ACL or T-arm). The manuscript does not close the gap of missing structural information, which would allow to directly characterize the mechanism of tRNA recognition by human DUS enzymes. However, there is a significant amount of new information presented in this manuscript – on an important topic that is understudied. In summary, the manuscript is timely and original - I recommend publication in ACS Central Science after the following points have been addressed.

Response: We thank the reviewer for their positive evaluation of our approach and findings. We agree that there are still some important questions left to answer, such as the structure of the DUS-tRNA catalytic complex, which we feel lies outside the scope of the current study. We are pursuing

this using our mechanism-based crosslinking strategy for a future report. Responses to specific issues can be found below.

Major issues

1. I was surprised to see that the DUS2 C116A mutants still seems to show some modification activity (Suppl. Fig. 3). I am aware that the detected activity is still lower than for the wild type protein, but it still seems very surprising that this mutant is able to convert U to D at all – could the authors discuss this finding and provide possible alternative residues that can take promote the modification reaction in the absence of the catalytic cysteine residue? Did the authors test other active site mutants?

Response: D formation by hDUS2 C116A was extremely low (roughly ~100-200 fold lower than in WT), consistent with the proposed catalytic mechanism for DUS proteins (PMID: 19139092). We have plotted the values on the same axis for comparison (see figure below). While the WT enzyme reaction reaches saturation at 30 min with a measured D/C ratio of ~0.02, in the C116A mutant we measure D/C = ~0.0001 at the same 30 min timepoint. The 0.0001 D/C value is also near the limit of detection in our LC-MS assay.

Given the extremely low activity we did not consider other residues in the active site as they are unlikely to be relevant for catalysis by the WT enzyme. It is also possible that protonation at C5 could occur through a non-enzymatic route in the C116A mutant.

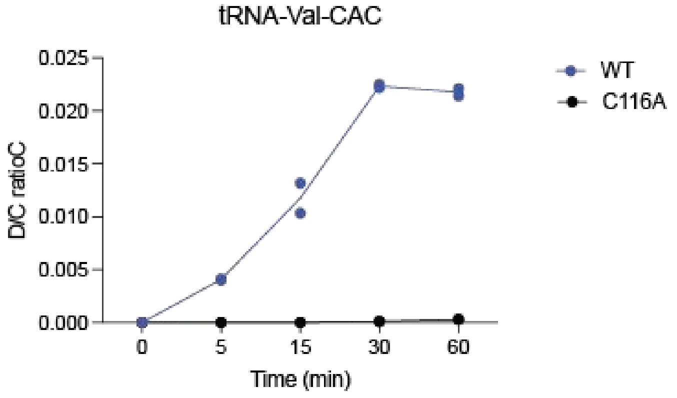

2. Suppl. Fig 4b, the gel of IVT BrUrd-Val tRNA clearly shows 2 lower bands, indicating other species of produced tRNA (3'truncations or not-properly folded tRNAs). Did the authors analyze all 11 produced tRNA and correlate the level of heterogenicity of IVT tRNA with the effect on crosslinking efficiency. It could mean that less stable tRNAs that have lower crosslinking efficiency (i.e. Tyr or Asn) are simply more heterogeneous. The quality of IVT tRNA preparations could influence conclusions made by authors. Did they consider synthesis of tRNAs or tRNAs fragments (i.e. without ACL) that would have only modified Br-Urd at position 20 instead of exchanging all Us? Modifying all U bases may have detrimental effect on tRNA folding and it would be necessary to more quantitatively assess the quality of the IVT tRNAs (e.g. Tm, DSL).

Response: We thank the reviewer for raising several excellent points. Densitometry analysis of IVT BrUrd-modified tRNA-Val-CAC (Supp. Fig. 4b) indicates that the fulllength product is the major product (>90%) in the sample. Therefore, full-length tRNA should be the primary contributor to the crosslinking adduct with hDUS2 in our assay. We have now also provided a gel showing the quality of all 11 BrUrd-modified tRNA substrates used for crosslinking (Supp. Fig. 9c), which demonstrates comparable homogeneity among these substrates. We also performed Tm analysis of IVT BrUrdmodified and unmodified tRNA-Val-CAC using CD spectroscopy (see below). Our analysis demonstrates very similar structural transitions indicating that the BrUrd modification is unlikely to grossly perturb structure within this tRNA.

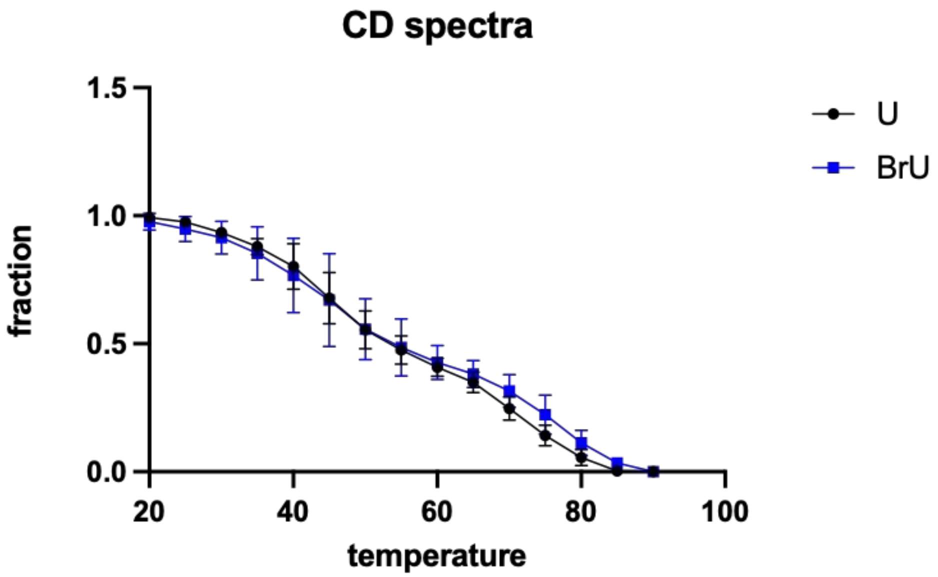

Importantly, we also considered that BrUrd modification may artificially impact hDUS2tRNA recognition and therefore we further studied hDUS2 enzymatic activity on unmodified IVT tRNAs using oligo LC-MS analysis (Fig. 4). We are of course aware that the IVT unmodified tRNAs are not the “native” substrate however it is commonplace to use these constructs in the field given the challenge of isolating biochemical quantities of native human tRNAs. Crosslinking efficiency and enzymatic modification efficiency showed a strong correlation, supporting the reliability of our crosslinking approach as a primary screening platform.

Regarding synthesis of a tRNA containing a single BrUrd modification on position 20 – we plan to investigate such constructs in our future studies, however given the inefficiencies and expense of solid-phase RNA oligo synthesis, IVT is a much more practical and efficient way to generate a panel of BrUrd-modified tRNAs, as was needed for our work.

3. Fig.2b and Suppl. Fig.5,– crosslinking reactions were performed with the substantial molar excess of tRNA (1uM or 10uM) to protein (200nM) content (5-fold or 50-fold excess of BrU-tRNA-Val). This result suggests that the crosslinking reaction has low efficiency and indicates that BrU-tRNA is indeed not properly folded (see point above) and could be improved by specific modification of only U20 base, targeted by hDUS2. A similar observation was made in BrU-tRNA-Val titration (Suppl. Fig.7), as the authors state on p.7: “Crosslinking yields did not exceed ~50%

despite using up to 75fold excess of BrU-tRNA-Val-CAC, which is likely due to instability of the enzyme during the reaction but could also reflect inefficiency in the crosslinking chemistry." Moreover, the upscaled experiment (Suppl. Fig. 8) for proteomics analysis have reached 95% crosslinking efficiency with the use of 2 µg of recombinant hDUS2 with 7.5 µg of BrUrdmodified tRNA-Val-CAC (3,75-fold excess of tRNA). Is it the same IVT BrU-tRNA-Val preparation? If not, it could mean that tRNA purity and folding varies between the preparation and should be clarified.

Response: There are many factors that are likely to contribute to crosslinking efficiency including (but not limited to) the stability and folding of enzyme and tRNA, recognition of the BrUrd-modified tRNA, and efficiency of the crosslinking chemistry. To maintain consistency, all IVT tRNAs were prepared following the same protocol outlined in the Supporting Information and refolded prior to usage.

Regarding the increase in crosslinking efficiency in the upscaled experiment (Supp. Fig. 8) this could be due to the higher concentration of enzyme used (700 nM enzyme in the upscaled experiment as compared to 200 nM in the small-scale experiments).

4. As the highest efficiency of crosslinking in Fig.3a-d was observed for tRNAGlu and tRNAVal, the optimal sequence recognition motif could be G18G19U20U20a – present in both tRNAs and not in the others tested. Similarly, the lack of dsRBD domain of hDUS2 doesn't abolish the crosslinking to both tRNAGlu and tRNAVal, indicating stronger interactions of the catalytic core with the G18G19U20U20a / G18G19U20 motif as the one present in tRNAGly and tRNALeu, which is efficiently modified by hDUS2 lacking the dsRBD (Fig. 3d, Suppl. Fig. 15). Moreover, the experiments with the modified oligonucleotides (shown on Suppl. Fig. 13), and with truncated tRNAs (Suppl.Fig. 14) indicate that substrate selection by hDUS2 requires tertiary tRNA structure determinants as deletion of T-arm or acceptor stem abolished crosslinking to hDUS2. Therefore, the "GU" motif seems not to be sufficient for governing the hDUS2 substrate selectivity. The authors could consider comparing the predicted 3D structures of all tested IVT tRNAs to conclude about DUS2-selective determinants in tRNAs. Foremost, I think the authors overstate the phrase "motif" and I would kindly ask the authors to down-tone this statements – on the one hand they show that whole domains of the tRNA are important and on the other hand they claim that the "motif" only consists of GU – this is inconsistent and confusing to the novice reader.

Response: There is no question that the dinucleotide "GU" alone is insufficient for hDUS2 recognition and modification on RNA. Our study clearly shows that this motif is only relevant within the D loop of a full-length or largely full-length tRNA. The phrase "GU motif" is used 2 times in our manuscript. Once in the abstract, and once in the discussion. In each instance, we are careful to reference this motif within the "tRNA tertiary fold" (Abstract) or within the "tRNA D loop" (Discussion), making clear that the dinucleotide is only functionally sufficient when it is within the appropriate context.

At the same time, our experiments with tRNA-Arg-ACG and tRNA-Val-CAC show clearly that the preceding G is required for modification and can be artificially installed at adjacent positions in the D loop to reprogram the register of modification. While G18G19U20U20a may be optimal, it is not required as many tRNAs that are hDUS2 substrates lack this sequence. We also interrogated

whether “G18G19U20” constituted the minimal required motif by generating tRNA-Val-CAC containing a G18A mutation (Supp. Fig. 47-48) and investigating its modification by hDUS2. Our data supports that only a single 5’ G is required.

5. Related to the slight overstatement about the identified “motif”, I would suggest to adjust the title – at the moment it is rather confusing and does not describe the major findings in the most clear way.

Response: We have adjusted the title to “Sequence- and structure-specific tRNA dihydrouridylation by hDUS2”

Minor points:

- Removal of the ASL doesn’t seem to influence the interaction between DUS2 and tRNA as shown by activity-based crosslinking experiments (Fig3e,f) and all tested tRNAs harbor the GU motif. I would ask the authors to comment on the observed large variety in crosslinking efficiency between different tRNA species (Fig.3a,b and Fig3.c,d)? - also see points above.

Response: Removing the ASL does modestly reduce crosslinking efficiency, even though this region is not strictly required (Fig. 3e, Supp. Fig. 14b). Generally, our data points towards the importance of the overall tertiary tRNA structure for hDUS2 recognition and modification, in particular the formation of the tRNA “elbow” where U20 is found and which is not thought to involve the ASL (PMID: 26771646). The differences in catalytic activity that we observe on different tRNAs could result from a number of factors. Sequence or structural variation could be involved – as the reviewer writes above, some of the best tRNA substrates (Val and Glu) contain G18G19U20U20a in the D loop. Further, these are all IVT tRNAs, and tRNA modifications could have varying degrees of importance for tRNA structure and hDUS2 recognition. Unfortunately, there are extremely few high-resolution structures of human tRNAs (either of endogenous fully modified tRNAs or IVT tRNAs), and therefore we hesitate to speculate on the nuances that small sequence variations may impart on tRNA structure and enzymetRNA recognition, in the absence of major effects (i.e. it is clear that G19U20 is a strict requirement). Instead, as we write in the Discussion, further elucidation of the basis of substrate preference will require structural studies which we believe lies outside of the scope of the current manuscript. We are currently pursuing such studies for future work.

- The authors claim that they have identified the minimal GU motif that governs the activity of hDUS2 enzyme. There are not enough direct experiments supporting this conclusion. The G19 is very conserved base in almost all human tRNA molecules. The lack of G preceding the modifiable U20 may lead to disrupted substrate recognition but does not influence the enzyme activity. Thus, the abstract sentence on p.2 should be rephrased: “We find that hDUS2 modifies U20 in the D loop of diverse tRNA substrates and identify a minimal GU motif within the tRNA tertiary fold required for directing its activity.” Furthermore, the paragraph on page 15 seems way too speculative – “G19 is known to Watson-Crick base pair with C56 in the T loop23, forming a conserved tertiary interaction at the tip of the tRNA “elbow”. It is likely that direct recognition of the G19:C56 pair in the context of this structure is responsible for setting the register of modification. Interestingly, mutational insertion of G at position 20 in either tRNA-ValCAC or tRNA-Arg-ACG enables modification by

hDUS2 at position 20a. One possible explanation for this behavior is that G20 pairs with C56 in place of G19 leaving the overall elbow structure unperturbed." – please rephrase.

Response: It seems that the reviewer is asking whether G19 is directly recognized by hDUS2 or whether its importance is related to its involvement in tRNA tertiary interactions, and also whether substrate recognition is distinct from catalytic activity. Regarding the latter, substrate recognition is a critical part of enzyme activity. Regarding the former, based on our data we are not able to differentiate between these two possibilities, however they are not mutually exclusive. It is true that G19 is universally conserved, and therefore our key question was how hDUS2 differentiates between modification at U20 and U20a (we established on all tRNAs studied that hDUS2 only modifies U20). The key experiments supporting our claim are the following (Fig. 4):

- Mutation of A20 to G20 in tRNA-Arg-ACG results in D modification at U20a, which is not modified in the WT tRNA
- Mutation of U20 to G20 in tRNA-Val-CAC results in D modification at U20a, which is not modified in the WT tRNA
- Mutation of U20 to C20 in tRNA-Val-CAC does not result in D formation at U20a.
    - Mutation of G18 to A18 in tRNA-Val-CAC does not abolish D formation on U20.

We have rephrased some of the sections requested by the reviewer to read as follows:

(Abstract p.2)

We find that hDUS2 exclusively modifies U20 across diverse tRNA substrates and identify a minimal GU sequence within the tRNA D loop that underlies selective substrate modification.

(Discussion p. 15) (as this is the Discussion section, it is customary to propose some possible hypothesis supported by the data)

What is the role of the preceding G19 residue in directing modification at U20 but not proximal U20a/U20b? G19 is known to Watson-Crick base pair with C56 in the T loop[23], forming a conserved tertiary interaction at the tip of the tRNA "elbow". We propose that direct recognition of G19U20 dinucleotide in the context of this structure is responsible for setting the register of modification. Alternatively, local unfolding of the G19:C56 pair could be involved in enzyme recognition. Interestingly, mutational insertion of G at position 20 in either tRNA-Val-CAC or tRNA-Arg-ACG enables modification by hDUS2 at position 20a, suggesting that the G20U20a dinucleotide in these mutant tRNAs is recognized similarly to the WT G19U20 dinucleotide.

- I would suggest to label the RNA bases as U20 etc. instead of U20.

- The sentence in the introduction on p.4 asks for an explanation WHY the authors did not follow up on the previous results for DUS3L - "Despite the proposed mechanistic similarity among DUS enzymes, we found that 5-FUrd did not react appreciably with human DUS enzymes other than DUS3L. In addition, we did not characterize the nature of the 5-FUrd-DUS3L crosslink nor did we

reconstitute DUS3L-RNA crosslinking in vitro.”. Did the authors try to reconstitute the DUS3L-RNA in vitro and failed?

Response: Our preliminary findings show that DUS3L is insoluble in bacterial expression systems, therefore we were unable to pursue the same in vitro studies as described in this manuscript for hDUS2. As we show in the manuscript, DUS3L expressed in HEK293T cells does crosslink in lysate with BrUrd-modified tRNA, indicating that our approach could be used for other DUS enzymes. We are currently pursuing other expression systems for *in vitro* studies of DUS3L, which we hope to report upon in the future.

- Suppl. Fig. 11 and 12 – it would be very interesting to identify the crosslinking U base in tRNAMet as it may not be the U16. It could also be considered as an artefact since the crosslinking efficiency is very low – please comment.

Response: We agree that identifying the crosslinked based would be interesting, however since crosslinking was low and was not accompanied by high levels of dihydrouridylation on the corresponding unmodified IVT substrate (as measured by LCMS, Supp. Fig. 12), we did not pursue it further.

- Suppl. Fig. 45 – please explain the double peak at the observed LC-MS analysis.

Response: RNase T1 digests after G residues, and based on the sequence of tRNAArg, there are two fragments generated with the same mass after RNase T1 digestion (UAACGp and AUCAGp). Since the extracted ion chromatography represents all the peaks identified as the same m/z value, it picks up both fragments. To distinguish these two peaks, we did MS2 analysis to confirm their identities (Supp. Fig. 46).

- Page 11 line 39 - typo “a similar same set of tRNAs”

Response: Thank you, we have fixed this.

Additional Questions:
Quality of experimental data, technical rigor: High

Significance to chemistry researchers in this and related fields: Top 5%

Broad interest to other researchers: High

Novelty: High

Is this research study suitable for media coverage or a First Reactions (a News & Views piece in the journal)?: No

Reviewer: 3

Recommendation: Publish in ACS Central Science after minor revisions noted.

Comments:
In this manuscript, Ji et al provide a series of experiments to test the substrate specificity of hDUS2. These approaches include mechanism-based crosslinking and oligonucleotide LC-MS/MS. Through their approach they were able to show the molecular determinants for substrate recognition and modification by hDUS2. Besides this, the authors further developed a screening approach to identify small molecule inhibitors of hDUS2, that is involved in human cancer. The manuscript is extremely well organized, very comprehensive and answers all the main questions raised in the initial hypothesis. Through the reading of the manuscript the reader is led to think about certain questions, as for example, how is substrate specificity of DUS2 in other tRNAs, or how can the authors ensure that BrUrd modification is not affecting the enzyme recognition. For all these questions, the authors provide answers with compelling evidence that are the result of the development and implementation of several very elegant strategies. The establishment of an oligonucleotide LC-MS platform to characterize the site of hDUS2 mediated formation on unmodified IVT tRNA substrate is another example of a very elegant methodology that can be applied to study many other tRNA modifications. In sum, this is a very good manuscript that, in my opinion, is mostly ready to be published. I only have 2 very minor remarks: - Fig 2b is not mentioned in the text

Response: We thank the reviewer for their positive feedback. We referenced Fig. 2b on page 7, line 6: "We observed the formation of a protein-RNA crosslink that was confirmed by RNase treatment and was not found with tRNA-Val-CAC containing canonical U residues (Fig. 2b, Supplementary Fig. 5)."

- Maybe the text following the question "How does hDUS2 select a single U residue among the multiple possible U substrates within the D loop?" could be improved, as the introduction of a question in the middle of the results section may be misleading. But this is more a matter of "taste" rather than a critique, and I leave it to the authors consideration.

Response: We thank the reviewer for this constructive comment and have modified the text in the following way:
"We next investigated the sequence determinants for selective tRNA modification by hDUS2 in the D loop. In particular, we were interested in the ability of hDUS2 to modify only U20 among multiple adjacent U residues (i.e. 20/20a/20b) as we observed for tRNA-Val-CAC, tRNA-Glu-TTC, and tRNA-Leu-CAA."

Additional Questions:
Quality of experimental data, technical rigor: Top 1%

Significance to chemistry researchers in this and related fields: Top 5%

Broad interest to other researchers: High

Novelty: High

Is this research study suitable for media coverage or a First Reactions (a News & Views piece in the journal)?: No

Reviewer: 4

Recommendation: Publish in ACS Central Science after minor revisions noted.

Comments:
Kleiner and coworkers describe the use of a novel chemical tool set to understand the selectivity of human uridine to dihydrouridine tRNA modifications catalyzed by the hDUS2 tRNA modification enzymes. Previous work has established the biological importance of hDUS2 modifications and also possible disease relevance. Less understood has been the selectivity of these enzymes and exact biological role of such modifications, and its clear that, even more broadly speaking, novel tools for understanding and characterizing tRNA modifications are needed. Kleiner uses a clever mechanism based cross linking assay to better understand hDUS2 activity. They identify the necessary selectivity requirements for modification and even demonstrate that the tool could prove useful for screening drug interactions with the enzyme. Overall, it is technically very well executed. One question that came up during reading was the surprising result that Afatinib increases activity of hDUS2. This is mentioned but not commented on and I think its worth a brief discussion of what might be going on.

Response: We thank the reviewer for this comment. We were also surprised by the increase in activity after afatanib treatment. We have amended the text to now read: "Surprisingly, Afatinib was found to modestly activate hDUS2 – this may occur through labeling of an allosteric cysteine or through non-covalent interactions."

Since we do not have further data, it seems premature to speculate beyond this statement. As we comment in the text, we believe that an unsubstituted acrylamide warhead (afatanib is the only compound we assayed with a substituted a,b-unsaturated amide) is necessary for inhibition of hDUS2.

Additional Questions:
Quality of experimental data, technical rigor: Top 5%

Significance to chemistry researchers in this and related fields: High

Broad interest to other researchers: High

Novelty: High

Is this research study suitable for media coverage or a First Reactions (a News & Views piece in the journal)?: No

oc-2023-01382r.R2

Name: Peer Review Information for "Sequence- and structure-specific tRNA dihydrouridylation by hDUS2"

Second Round of Reviewer Comments

Reviewer: 3

Comments to the Author

The authors have addressed all the reviewers comments appropriately. This revised version confirms the high quality of this manuscript and is ready to be fully accepted for publication.

Reviewer: 4

Comments to the Author

The authors have addressed my comments appropriately

Reviewer: 2

Comments to the Author

In this manuscript the authors study human dihydrouridine synthase 2 (hDUS2) and investigate its enzymatic activity and substrate specificity using novel activity-based probes.

I had time to re-evaluate the revised version of the manuscript. The authors have addressed all my questions. Even if some the answers don't full resolve this raised issues, the responses provide a reasonable level of explanation. Hence, I support publication of the revised manuscript in ACS Central.

Author's Response to Peer Review Comments:

Dear Editor,

Thank you for overseeing the review of our manuscript and for the positive decision on publication. We have made the requested editorial formatting changes to the manuscript and SI and revised files are attached.

Best,

Ralph
